# Bile Acids Reduce Endocytosis of High-Density Lipoprotein (HDL) in HepG2 Cells

**DOI:** 10.1371/journal.pone.0102026

**Published:** 2014-07-10

**Authors:** Clemens Röhrl, Karin Eigner, Stefanie Fruhwürth, Herbert Stangl

**Affiliations:** Department of Medical Chemistry, Center for Pathobiochemistry and Genetics, Medical University of Vienna, Vienna, Austria; Tohoku University, Japan

## Abstract

High-density lipoprotein (HDL) transports lipids to hepatic cells and the majority of HDL-associated cholesterol is destined for biliary excretion. Cholesterol is excreted into the bile directly or after conversion to bile acids, which are also present in the plasma as they are effectively reabsorbed through the enterohepatic cycle. Here, we provide evidence that bile acids affect HDL endocytosis. Using fluorescent and radiolabeled HDL, we show that HDL endocytosis was reduced in the presence of high concentrations of taurocholate, a natural non-cell-permeable bile acid, in human hepatic HepG2 and HuH7 cells. In contrast, selective cholesteryl-ester (CE) uptake was increased. Taurocholate exerted these effects extracellularly and independently of HDL modification, cell membrane perturbation or blocking of endocytic trafficking. Instead, this reduction of endocytosis and increase in selective uptake was dependent on SR-BI. In addition, cell-permeable bile acids reduced HDL endocytosis by farnesoid X receptor (FXR) activation: chenodeoxycholate and the non-steroidal FXR agonist GW4064 reduced HDL endocytosis, whereas selective CE uptake was unaltered. Reduced HDL endocytosis by FXR activation was independent of SR-BI and was likely mediated by impaired expression of the scavenger receptor cluster of differentiation 36 (CD36). Taken together we have shown that bile acids reduce HDL endocytosis by transcriptional and non-transcriptional mechanisms. Further, we suggest that HDL endocytosis and selective lipid uptake are not necessarily tightly linked to each other.

## Introduction

Cholesterol is an essential constituent of cell membranes, modulates cell signaling and is a precursor for steroid hormone and bile acid synthesis. However, excess cholesterol accumulation in peripheral cells including macrophages can trigger atherosclerosis. Mammalian cells are not capable of catabolizing cholesterol and therefore excretion via the bile is the only way to remove excess cholesterol from the body. High-density lipoprotein (HDL) is a main carrier of cholesterol in the circulation and transports excess peripheral cholesterol to the liver for biliary excretion. This process is termed reverse cholesterol transport (RCT) and is thought to be an important atheroprotective property of HDL [1], [2].

For biliary cholesterol excretion, HDL-cholesterol has to be transported to hepatocytes first. Two main pathways facilitate lipid transfer: First, HDL cholesterol is transferred to cells by selective lipid uptake, which involves HDL binding to the scavenger receptor class B, type I (SR-BI) and selective transfer of HDL associated lipids [3], [4]. Second, HDL is endocytosed and lipids are exchanged during intracellular trafficking of HDL [5], [6], [7]. The importance of selective lipid uptake in maintaining cholesterol homeostasis is well established and the mechanisms regulating SR-BI expression and function are under extensive investigations [8]. In contrast, the contribution of HDL endocytosis to the maintenance of cholesterol homeostasis is controversially discussed [9]. Additionally, the analysis of receptors and mechanisms regulating HDL endocytosis is insufficiently addressed. An exception is the work of the lab of Laurent Martinez, who identified the apolipoprotein A-I cell surface receptor F_1_-ATPase and the nucleotide receptor P_2_Y_13_ as potent regulators for HDL endocytosis in hepatic cells [10]. Extracellular ADP generated by F_1_-ATPase stimulates the purinergic receptor P_2_Y_13_, which in turn activates HDL endocytosis by a low affinity HDL receptor that remains to be characterized. Indeed, HDL uptake into the liver as well as reverse cholesterol transport is decreased in mice lacking P_2_Y_13_ [11]. More recently it was shown that pharmacologic P_2_Y_13_ activation increased hepatic HDL uptake and augmented development of atherosclerosis in apoE^−/−^ mice [12].

After the transfer of HDL-cholesterol to hepatocytes, cholesterol is secreted into the bile either directly or indirectly after conversion to bile acids [13]. Due to the highly efficient enterohepatic cycle the majority of bile acids is reabsorbed into the circulation [14]. Given the fact that HDL is a main determinant of bile acid secretion [15] and that bile acids are also present in plasma, we asked if bile acids regulate HDL endocytosis. The existence of such a mechanism would constitute a feedback mechanism to regulate biliary secretion via HDL. In this study we aimed to analyze, if bile acids are capable of modifying HDL endocytosis. On the one hand, bile acids may act extracellularly, for instance by activating lipases or functioning as detergents. On the other hand, bile acids are taken up into hepatocytes and act as transcriptional activators for the farnesoid X receptor (FXR) [16]. In this manuscript we show that bile acids indeed regulate HDL endocytosis in human hepatic cell lines by exerting extracellular as well as transcriptional effects.

## Experimental Procedures

### Cell culture

Cells were cultivated under standard conditions. HepG2 cells (ATCC: HB-8065; Manassas, VA, USA) were grown in MEM supplemented with 10% FBS, 1% penicillin/streptomycin, and 1% non-essential amino acids (all from PAA, Pasching, Austria). HuH7 cells (ATCC: JCRB-0403) were maintained in DMEM containing 10% FBS and 1% penicillin/streptomycin. Lipoprotein deficient serum (lpds) was prepared from FBS as described [17]. All bile acids used and GW4064 were from Sigma (St. Louis, MO, USA).

Cells were seeded on day 0 in growth media and were treated on day 2. On the one hand, cells were incubated with bile acids in MEM containing 2 mg/ml fatty acid-free BSA (faf-BSA; PAA) for 1 hour and HDL uptake was analyzed simultaneously. On the other hand, cells were treated with bile acids or GW4064 in MEM containing 10% lpds for 24 hours followed by analysis of HDL uptake for 1 hour in MEM containing 2 mg/ml faf-BSA.

### SR-BI knock-down cells

HepG2 cells were seeded in 24-well plates. Lentiviral transduction was performed using 8 µg/ml of polybrene and 2*10^5^ TU of shRNA lentiviral transduction particles targeting SR-BI (SHCLNV, TRCN0000056963, MISSION Lentiviral Transduction Particles; Sigma) or scrambled control (SHC002V, MISSION pLKO.1-puro Non-Mammalian shRNA Control Transduction Particles; Sigma). Cells were centrifuged (30°C, 1300 g, 90 min) and were selected two days after transduction with medium containing 2 µg/ml Puromycin (Life Technologies Carlsbad, CA, US).

### Lipoprotein isolation and labeling procedures

LDL and HDL were recovered from human plasma by serial ultracentrifugation at a density of 1.07 and 1.21 g/ml, respectively [18]. Lipoproteins were routinely analyzed for their apolipoprotein content by SDS-gel electrophoresis. To fluorescently label HDL and LDL, the apolipoprotein part was covalently linked to Alexa^488^ or Alexa^568^ as described [6].

Radiolabeling of HDL at its apolipoprotein part with sodium ^125^iodide (^125^I; Hartmann Analytic, Göttingen, Germany) was performed using the Pierce IODO-BEADS reagent kit (Thermo Scientific, Rockford, IL, USA). HDL was purified from unincorporated label using gel filtration. HDL double-labeled in its apolipoproteins and lipid moiety (^125^I/^3^H-CE-HDL) was performed as follows: 100 µCi [Cholesteryl-1,2 -^3^H(N)]-oleate (Perkin Elmer, Waltham, MA, USA) were evaporated under nitrogen in a glass tube and resuspended in 50 µl DMSO. HDL (1 mg/450 µl PBS) was added followed by incubation in a rocking water bath at 40°C for 2 hours. Afterwards, iodination and purification was performed as described above. Transferrin was purchased from Sigma and labeled with Alexa^488^ as described for HDL.

### Uptake experiments with fluorescently labeled lipoproteins and transferrin

Cells seeded on cover-slides were incubated with 50 µg/ml HDL-Alexa^488^, LDL-Alexa^568^ or 20 µg/ml transferrin-Alexa^488^ diluted in MEM containing 2 mg/ml faf-BSA at 37°C for 1 hour. Cells were washed and fixed in 4% formaldehyde in PBS at 4°C for 30 minutes. Samples were counterstained with DAPI, washed, mounted and visualized with an Axiovert microscope (Zeiss, Jena, Germany).

### Uptake experiments with radiolabeled HDL

Cells were incubated with 20 µg/ml ^125^I-HDL or ^125^I/^3^H-CE-HDL (∼600 cpm/ng for ^125^I and ∼800 cpm/ng for ^3^H-CE) in MEM with 2 mg/ml faf-BSA at 37°C for 1 hour. A 40-fold excess of unlabeled HDL was added to every forth data point. Media were recovered and cell monolayers were washed twice with cold Tris HCl (pH = 7.4), 0.9% NaCl and 0.2% BSA and twice without BSA. Cells were lyzed with 0.1 M NaOH. Radioactivity was determined using a γ-counter for ^125^I-HDL or a β-counter for ^125^I/^3^H-CE-HDL. Specific cell association was calculated by subtracting the amount of radioactivity detected with a 40-fold excess from total activity measured. Cell protein was quantitated using the Bradford Method (Biorad, Vienna, Austria) and HDL uptake was expressed as ng HDL per mg cell protein. Selective cholesteryl ester uptake was calculated by subtracting ^125^I-HDL uptake from ^3^H-CE-HDL uptake.

To distinguish between HDL binding and uptake, cell surface-bound HDL was displaced with a 100-fold excess of unlabelled HDL in media containing 2 mg/ml fafBSA and 10 mM Hepes at 4°C for 2 hours.

For HDL degradation analysis, media were collected after the incubation with ^125^I-HDL and proteins were precipitated using 50% TCA. The supernatant was extracted with chloroform, oxidized with 30% hydrogen peroxide and counted to determine the amount of acid-soluble material formed by the cells [19].

### Cytotoxicity

Cytotoxicity was analyzed by measuring release of lactate dehydrogenase (LDH) into the media. Cell culture supernatants were assayed for LDH activity by addition of pyruvate and NADH (both from Sigma; final concentration: 1 mM and 0.2 mM, respectively). Turnover of NADH was measured photometrically at 340 nm.

### Filipin Staining

Cells were seeded on cover-slips and after bile acid treatment they were fixed with 4% formaldehyde in PBS at 4°C for 30 minutes. Samples were stained with 50 µg/ml Filipin III (Sigma) diluted in PBS containing 10% lpds at RT for 30 minutes. Cells were washed, mounted and visualized with an Axiovert microscope (Zeiss).

### FPLC

HDL size was analyzed using an ÄKTA fast protein liquid chromatography (FPLC) system (GE Healthcare, Fairfield, CT, USA) equipped with a superpose-6 column at a flow rate of 0.1 ml/min. HDL elution was continuously monitored by measuring protein concentration at 280 nm.

### Extracellular ATP hydrolysis

ATP is secreted by hepatic cells under physiological conditions [10]. HepG2 cells were seeded in 24-well plates on day 0 and incubated with media containing 10% lpds on day 2. On day 3, cells were re-fed with media containing 2 mg/ml faf-BSA in the presence or absence of 1 mM taurocholate. The exchange of cell culture media triggers ATP release [11]. Aliquots of the supernatant were collected after 10, 30 and 60 minutes and ATP hydrolysis was measured as a decrease in extracellular ATP by luminescence using the ATP-lite kit (Perkin Elmer).

### Gene expression analysis

RNA was isolated using the RNeasy Plus Micro Kit (Qiagen, Düsseldorf, Germany) and cDNA was synthesized from 2 µg RNA. qRT-PCR was performed using the following TaqMan probes (Life Technologies): GAPDH (Hs99999905_m1), SHP (Hs00222677_m1), SR-BI (Hs00969821_m1), CD36 (Hs01567185_m1), and CEL (Hs00426932_m1). The expression levels of genes of interest were normalized to GAPDH expression levels.

### Western blot analysis

Proteins were isolated and equal amounts were separated by SDS-PAGE and transferred to nitrocellulose membranes (Sigma). After blocking, membranes were incubated with primary antibodies (anti-SR-BI, BD Biosciences 610882; anti-β-actin, Abcam ab8229; anti-CD36, Cayman 100011) at 4°C over-night. Membranes were then incubated with the appropriate horseradish peroxidase-coupled secondary antibodies followed by detection using the Super Signal chemiluminescence system (Thermo Scientific) and a Chemilmager 4440 (Biozym, Oldendorf, Germany).

### Fatty-acid uptake

^3^H-oleic acid (Perkin Elmer) was bound to faf-BSA as described [20]. HepG2 cells were seeded in 12-well plates on day 0 and treated with CDCA or GW4064 on day 2. On day 3, cells were washed twice with warm PBS and incubated with 170 µM ^3^H-oleic acid (0.5 mCi/mmol) for 2, 5 and 10 minutes. Afterwards, cells were washed twice with ice-cold PBS containing 2 mg/ml BSA and twice with PBS without BSA. Cells were lyzed with 0.1 M NaOH, radioactivity was determined using a β-counter and data were normalized to cell protein, as determined by Bradford assay.

### Quantification of fluorescence images and statistics

Fluorescence images were quantified using ImageJ 1.47v (NIH, Bethesda, MA, USA). At least 50 cells were analyzed for each experiment. Statistical analysis was performed using GraphPad Prism v4.00 (GraphPad Softerware Inc., La Jolla, CA, USA). Two-sided t-tests or ANOVA were used to compare two or more groups, respectively. Data are depicted as mean +/− SD. * indicates p≤0.05 and ** indicates p≤0.01. For experiments using fluorescence microscopy, representative images from at least three independent experiments are shown.

## Results

To study extracellular effects of bile-acids on HDL endocytosis, we used human hepatic HepG2 cells treated with taurocholate. Although HepG2 cell types are derived from hepatocarcinoma cells they are frequently used in lipoprotein research because they maintain certain key features of hepatocytes such as apolipoprotein secretion. Taurocholate is a naturally occurring charged bile acid that is not taken up into hepatic cells *in-vitro*, because the expression of its transporter, the Na^+^-taurocholic acid cotransporting polypeptide (NTCP), is rapidly down-regulated after taking primary hepatocytes in culture [21]. As a second approach, we used chenodeoxycholate (CDCA) to examine transcriptional effects, as CDCA is lipophilic and is taken up into cells independently of a specific transporter.

**Figure 1:**
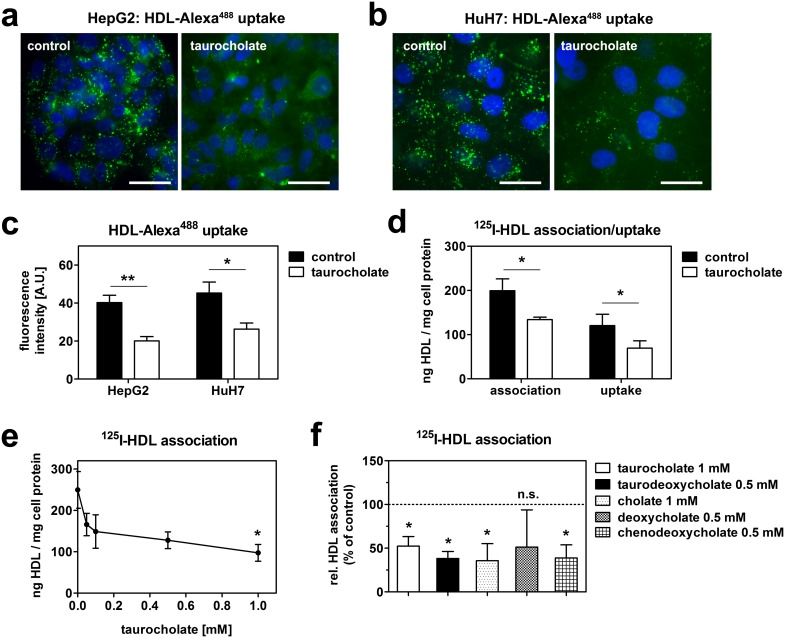
Bile acids reduce HDL endocytosis. HepG2 (a) and HuH7 (b) cells were incubated with 50 µg/ml HDL-Alexa^488^ with or without 1 mM taurocholate at 37°C for 1 hour. Cells were fixed, counterstained with DAPI and imaged. Green: HDL; blue: nucleus; bar = 10 µm. Representative images of 3 independent experiments are shown. (c) Quantification of fluorescence intensities of (a) and (b). (d) HepG2 cells were incubated in media containing 20 µg/ml ^125^I-HDL with or without 1 mM taurocholate at 37°C for 1 hour. Uptake was determined after displacing cell surface bound HDL by a 100-fold excess at 4°C for 1 hour (n = 3). (e) Cells were incubated with 20 µg/ml ^125^I-HDL with the indicated concentrations of taurocholate for 1 hour (n = 3). (f) Cells were incubated with 20 µg/ml ^125^I-HDL together with different bile acids for 1 hour (n = 3). Of note taurodeoxycholate, deoxycholate and chenodeoxycholate were cytotoxic at 1 mM and were therefore used at 0.5 mM.

HepG2 cells were incubated with HDL fluorescently labeled at its apolipoprotein moiety (HDL-Alexa^488^) with or without 1 mM taurocholate for one hour (Fig. 1a). In control cells incubated with fluorescent HDL without taurocholate, a vesicular staining pattern was apparent. Previously, we have identified these cellular compartments as multivesicular endosomes [6]. This endosomal staining was markedly reduced in taurocholate treated cells, indicating reduced HDL endocytosis. Similarly, HDL endocytosis was reduced by taurocholate treatment in HuH7 cells, another human hepatic cell line (Fig. 1b). Quantification of fluorescent signals revealed a reduction in HDL staining by approximately 50% in both cell lines (Fig. 1c). As an independent approach to quantify the consequence of taurocholate on HDL endocytosis, we utilized HDL radiolabeled at its apolipoproteins (^125^I-HDL). Specific HDL cell association (i.e. binding plus uptake) was likewise decreased in HepG2 cells when taurocholate was present in the media. When cell surface-bound HDL was displaced at 4°C, the remaining intracellular activity was still significantly reduced, confirming reduced HDL endocytosis upon taurocholate treatment (Fig. 1d). Of note, HDL degradation was merely detectable and did not significantly differ between control and taurocholate treated cells (5.7+/−1.8 ng/h vs 3.4+/−2.5 ng/h; p = 0.3).

The effect of taurocholate on HDL cell association was dose-dependent (Fig. 1e). However, statistical significance was only reached when taurocholate was added at a concentration of 1 mM. To exclude an effect specific for taurocholate, several other bile acid species were tested. Taurodeoxycholate, cholate and chenodeoxycholate had comparable effects on HDL endocytosis in HepG2 cells. Although not significant, HDL association also tended to be lowered by deoxycholate (Fig. 1f).

**Figure 2:**
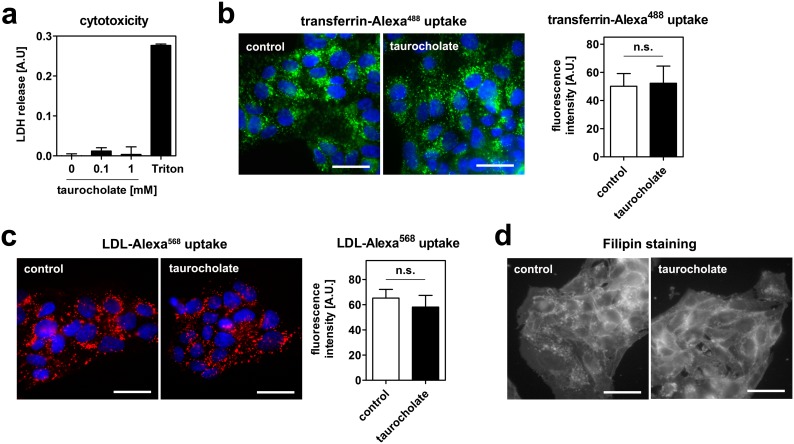
Taurocholate neither exerts cytotoxic effects, nor inhibits transferrin or LDL endocytosis in HepG2 cells. (a) Cells were incubated with the indicated concentrations of taurocholate for 1 hour. No release of LDH into the cell culture supernatant was detected. 0.1% Triton-X100 was used as a positive control. (b) Cells were incubated with 20 µg/ml transferrin-Alexa^488^ (b) or 50 µg/ml LDL-Alexa^568^ (c) with or without 1 mM taurocholate at 37°C for 1 hour. Cells were fixed, counterstained with DAPI and imaged. Green: transferrin; red: LDL; blue: nucleus; bar = 10 µm. Neither transferrin nor LDL uptake were altered. Quantifications of fluorescent signals are depicted next to the images. (d) Cells were incubated with or without 1 mM taurocholate for 1 hour. Cells were fixed, stained with Filipin and imaged. Bar = 10 µm. Representative images of 3 independent experiments are shown.

High bile acid concentrations may exert cytotoxic effects or affect cell membrane integrity by acting as detergents. To exclude the interference of cytotoxic effect with the experiments, we measured LDH release into the cell culture media after taurocholate treatment. No increase in LDH release was observed (Fig. 2a), suggesting that the taurocholate concentrations used do not exert acute cytotoxic effects in our experimental setup. Moreover, the endocytosis of transferrin was unaltered upon taurocholate treatment, indicating functional endocytosis (Fig. 2b). Importantly, taurocholate did also not interfere with the uptake of LDL (Fig. 2c). Finally, Filipin staining revealed no apparent alteration in free cholesterol distribution (Fig. 2d), suggesting that taurocholate does not extract membrane cholesterol from cells. Taken together, bile acids reduce endocytosis specific for HDL without exerting apparent adverse effect on the cells.

**Figure 3:**
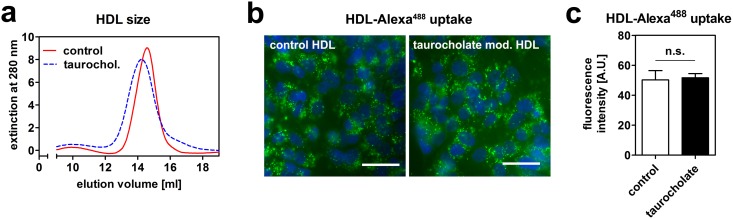
Modification of HDL by taurocholate does not alter endocytosis. (a) HDL was incubated with or without 1 mM taurocholate in media in the absence of cells for 1 hour. HDL size was then analyzed by size exclusion chromatography. HDL incubated with taurocholate is eluted earlier, indicating increased size. (b) HDL-Alexa^488^ was incubated with or without 1 mM taurocholate in media in the absence of cells for 1 hour. Free taurocholate was then removed using gel filtration and HepG2 cells were incubated with this modified HDL-Alexa^488^ for 1 hour. Cells were fixed, counterstained with DAPI and imaged. (c) Quantification of fluorescence intensities from (b); n = 3. Green: HDL; blue: nucleus; bar = 10 µm.

Next we tested, if this reduction in HDL endocytosis is due to modification of HDL by bile acids. When HDL was incubated with taurocholate in the absence of cells, HDL size increased as shown by size exclusion chromatography (Fig. 3a). This is presumably due to incorporation of bile acids into the HDL particle. As a next step, fluorescently labeled HDL was again incubated with taurocholate in the absence of cells and afterwards purified from unbound taurocholate. When HepG2 cells were incubated with this modified HDL or unmodified HDL, no difference was observed in HDL uptake (Fig. 3b, c). These data indicate that bile acids reduce HDL endocytosis independently of HDL modifications.

**Figure 4:**
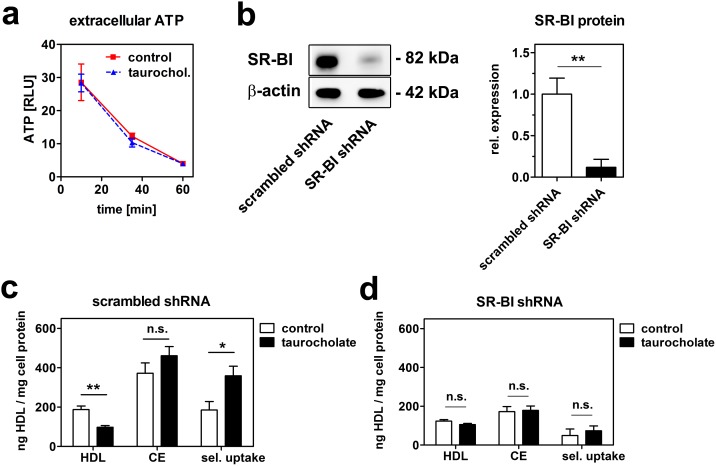
Taurocholate reduces HDL endocytosis SR-BI-dependently. (a) HepG2 cells were incubated with or without 1 mM taurocholate and ATP hydrolysis was measured as a decrease in extracellular ATP. One representative experiment out of three independent experiments is shown. (b) SR-BI knockdown efficiency in HepG2 cells transfected with scrambled shRNA and HepG2 cells transfected with SR-BI shRNA (n = 3). Selective lipid uptake analysis using double labeled ^125^I/^3^H-CE-HDL in scrambled control (c) or SR-BI knockdown (d) HepG2 cells (n = 3). Selective cholesteryl-ester uptake was calculated by subtracting ^125^I-HDL uptake from ^3^H-CE-HDL uptake.

An extracellular key regulator of HDL endocytosis is the ectopically expressed cell surface F_1_-ATPase. This enzyme is capable of hydrolysing extracellular ATP to ADP. ADP in turn activates the purinergic receptor P_2_Y_13_, which induces HDL endocytosis [10], [22]. Accordingly we analyzed, if taurocholate treatment alters the activity of F_1_-ATPase by measuring the hydrolysis of extracellular ATP. However, ATP hydrolysis was unaltered in the presence of taurocholate (Fig. 4a), suggesting that taurocholate does not influence the activity of extracellular ATPases.

To analyze a potential contribution of SR-BI to the reduction of HDL endocytosis, we performed experiments in HepG2 cells where SR-BI expression was reduced to 10% by lentiviral shRNA knockdown (Fig. 4b). HDL association experiments were performed using HDL particles double labeled in the apolipoprotein and lipid moiety (^125^I/^3^H-CE-HDL). In control cells transfected with scrambled shRNA, HDL holo-particle association (as measured by ^125^I activity) was reduced by taurocholate, whereas cholesteryl-ester (CE; measured by ^3^H activity) association was slightly increased (Fig. 4c). This resulted in a 2-fold increase of selective lipid uptake (calculated as CE minus HDL cell association). In SR-BI knockdown cells, association of HDL, CE and selective uptake were decreased compared to control cells. However, taurocholate treatment did not alter any of these parameters (Fig. 4d). These data suggest that the presence of bile acids in the cell culture medium reduces HDL endocytosis, but increases the effectiveness of selective CE uptake in hepatic cells by processes dependent on SR-BI.

After having shown that bile acids exert extracellular effects on HDL endocytosis, we analyzed if bile acids also alter HDL endocytosis via FXR, which is an essential regulator of cholesterol homeostasis [23]. We thus examined the consequences of FXR activation by bile acids on HDL endocytosis using CDCA. As CDCA may also exert FXR-independent effects, we additionally used the synthetic nonsteroidal FXR-specific agonist GW4064.

**Figure 5:**
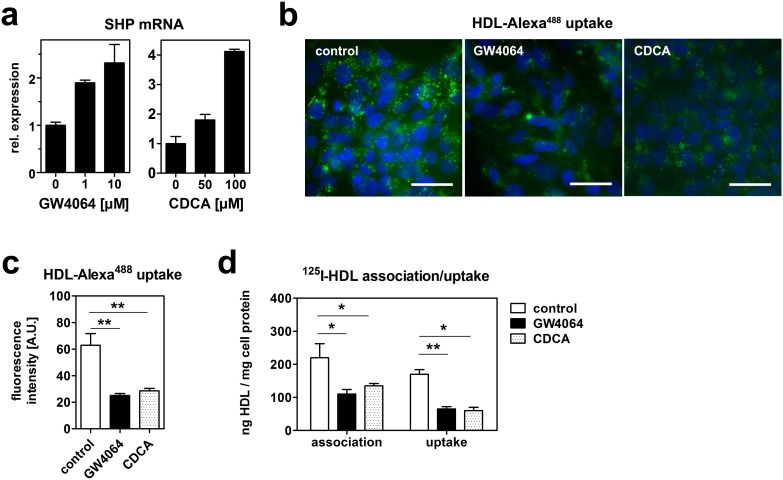
Bile acids and a non-steroidal FXR agonist reduce HDL endocytosis. (a) HepG2 cells were treated with the indicated concentrations of GW4064 or chenodeoxycholate (CDCA) in media containing lipoprotein-deficient serum (lpds) for 24 hours. Gene expression was analyzed by qRT-PCR and expression levels were normalized to GAPDH expression (n = 2). The increase in SHP mRNA indicates FXR activation. (b) HepG2 cells were incubated with 10 µM GW4064 or 100 µM CDCA in media containing lpds for 24 hours. Cells were then incubated with 50 µg/ml HDL-Alexa^488^ for 1 hour. Cells were fixed, counterstained with DAPI and imaged. Green: HDL; blue: nucleus; bar = 10 µm. (c) Quantification of fluorescence intensities of (b). (d) HepG2 cells were incubated with 10 µM GW4064 or 100 µM CDCA in media containing lpds for 24 hours. Cells were then incubated with 20 µg/ml ^125^I-HDL for 1 hour. Uptake was determined after displacing cell surface bound HDL by a 100-fold excess at 4°C for 1 hour (n = 3).

HepG2 cells were treated with GW4064 or CDCA in media containing lipoprotein-deficient serum (lpds) for 24 hours. FXR was activated as monitored by a dose-dependent increase in the expression of the small heterodimer partner (SHP), an established transcriptional FXR target gene (Fig. 5a). After incubation with 10 µM GW4064 or 100 µM CDCA, HDL endocytosis was analyzed by incubation with HDL-Alexa^488^ for one hour. Treatment with both FXR agonists led to a comparable decrease of HDL endocytosis (Fig. 5b, c). Subsequently, HDL cell association and uptake was quantified using ^125^I-HDL. Both GW4064 and CDCA reduced specific cell association of HDL by approximately 50%. This reduction in cell association was accompanied by a significant reduction in HDL uptake (Fig. 5d).

**Figure 6:**
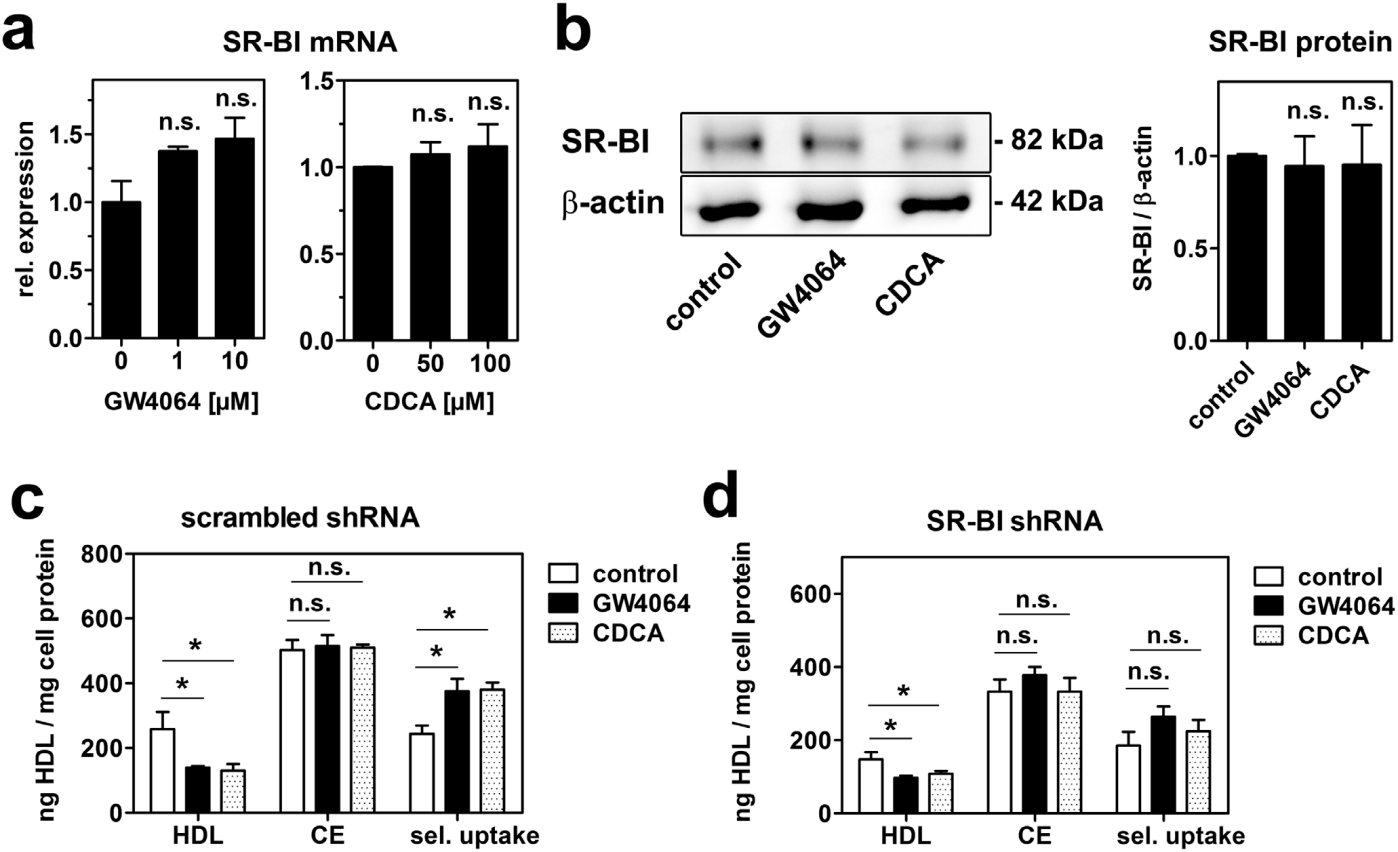
GW4064 and CDCA reduce HDL endocytosis independently of SR-BI. (a) HepG2 cells were treated with the indicated concentrations of GW4064 or chenodeoxycholate (CDCA) in media containing lipoprotein-deficient serum (lpds) for 24 hours and gene expression was analyzed by qRT-PCR (n = 3). (b) Cells were incubated with 10 µM GW4064 or 100 µM CDCA in media containing lpds for 24 hrs and protein expression was determined by western blot analysis and results were quantitated by densitometry (n = 3). HepG2 cells transfected with scrambled shRNA (c) or SR-BI shRNA (d) were incubated with 10 µM GW4064 or 100 µM CDCA in media containing lpds for 24 hours. Cells were then incubated with 20 µg/ml double labeled ^125^I/^3^H-CE-HDL for 1 hr. Selective cholesteryl-ester uptake was calculated by subtracting ^125^I-HDL uptake from ^3^H-CE-HDL uptake (n = 3).

Reports on positive as well as negative regulation of SR-BI by FXR are available [24], [25], [26]. Thus, SR-BI expression was studied after treatment with GW4064 or CDCA. SR-BI mRNA tended to increase dose-dependently with both FXR agonists (Fig. 6a). However, these effects did not reach statistical significance. SR-BI protein was unaltered after treatment with GW4064 or CDCA (Fig. 6b). To further clarify, if SR-BI is involved in the observed reduction of HDL endocytosis, cell association of ^125^I/^3^H-CE-HDL was analyzed in control and SR-BI knockdown cells. FXR activation by both CDCA and GW4064 reduced HDL association in control cells (Fig. 6c) as well as in SR-BI knockdown cells (Fig. 6d). CE uptake was unaltered leading to an increase of selective uptake in control cells, which was diminished in SR-BI knockdown cells. These data suggest that bile acids, besides acting extracellularly via SR-BI, reduce HDL endocytosis by FXR activation independently of SR-BI.

**Figure 7:**
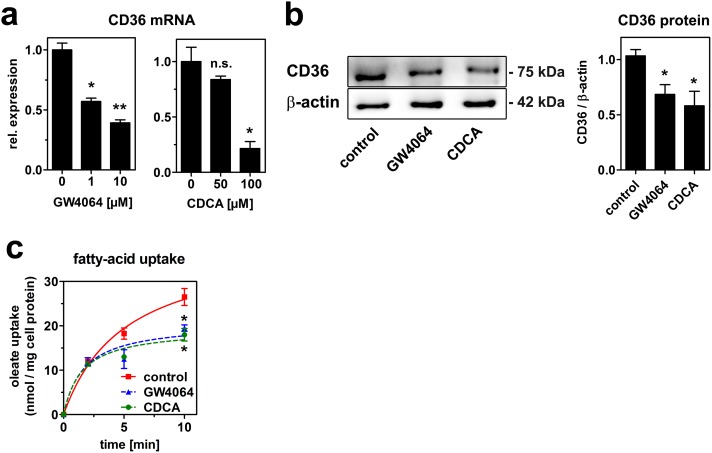
GW4064 and CDCA reduce CD36 expression and function. (a) HepG2 cells were treated with the indicated concentrations of GW4064 or chenodeoxycholate (CDCA) in media containing lipoprotein-deficient serum (lpds) for 24 hours and gene expression was analyzed by qRT-PCR (n = 3). (b) Cells were incubated with 10 µM GW4064 or 100 µM CDCA in media containing lpds for 24 hrs and protein expression was determined by western blot analysis and results were quantitated by densitometry (n = 3). (c) Fatty-acid uptake was determined after treatment with 10 µM GW4064 or 100 µM CDCA as described in the methods section (n = 3).

As an alternative receptor mediating the reduction in HDL endocytosis, we studied the expression of CD36. This receptor was initially identified as a transporter for fatty-acids and oxidized lipoproteins, and was recently described to mediate uptake of native HDL [27]. CD36 mRNA expression decreased dose-dependently by treatment with both FXR agonists (Fig. 7a). This reduction in mRNA expression translated into reduced CD36 protein expression (Fig. 7b). Further, fatty-acid uptake in response to treatment with CDCA and GW4064 was measured to test, if the reduction in CD36 is functional. Indeed, FXR activation reduced fatty-acid uptake significantly (Fig. 7c). Taken together, bile acids reduce HDL endocytosis by transcriptional and non-transcriptional effects. The latter are dependent on SR-BI, whereas the transcriptional effects are independent of SR-BI and might involve CD36.

## Discussion

HDL is a major determinant of bile acid secretion. Here we show that bile acids reduce HDL endocytosis in hepatic cells *in-vitro*, which might constitute a feedback mechanism for biliary cholesterol secretion *in-vivo*.

The presence of a panel of different bile acids in the media significantly reduced HDL endocytosis in HepG2 and HuH7 cells (Fig. 1). These effects were independent of altered receptor transcription, as taurocholate is not transported into tissue culture cells. Indeed, mRNA expression of SR-BI, CD36 or carboxyl-ester lipase (CEL) was unaltered after taurocholate treatment (data not shown). A key regulator of HDL endocytosis is the ectopically expressed cell surface F_1_-ATPase. This enzyme is capable of hydrolysing extracellular ATP to ADP. ADP in turn activates the purinergic receptor P_2_Y_13_, which induces HDL endocytosis [10], [22]. Accordingly we analyzed, if taurocholate treatment alters the activity of F_1_-ATPase by measuring the hydrolysis of extracellular ATP. However, ATP hydrolysis was unaltered in the presence of taurocholate (Fig. 4a). Of note, ATP hydrolysis is not a specific feature of F_1_-ATPase, as other ecto-ATPases contribute to extracellular ATP hydrolysis as well [28]. Therefore, additionally experiments would be necessary to definitely rule out a role of this pathway. However, our data suggest that bile acids do no alter HDL endocytosis via the F_1_-ATPase and the nucleotide receptor P_2_Y_13_ pathway.

In portal blood, bile-acid concentrations of 60 µM are measured in the postprandial state in men [29]. For taurocholate, 1 mM was used, which is beyond physiologic concentrations. Of note, we also observed a reduction in HDL endocytosis at lower concentrations, but these effects were not statistically significant (Fig. 1e). Therefore, 1 mM taurocholate was used for experiments. At this concentration, we could exclude acute cytotoxicity and extraction of membrane cholesterol from cells (Fig. 2a, d). Further, taurocholate did not impair endocytic trafficking, as shown by intact transferrin and LDL uptake (Fig. 2b, c). Thus, the effect on reduced endocytosis was specific for HDL. Moreover, bile acids did not interfere with HDL integrity (Fig. 3). If the extracellular effect of bile acids on HDL endocytosis is physiologically relevant remains to be investigated. It is interesting to hypothesize that extracellular and intracellular mechanisms cooperate to regulate HDL endocytosis by bile-acids *in-vivo.*

Despite reduced HDL endocytosis, selective lipid uptake was increased by taurocholate treatment (Fig. 4). This increase might be rationalized by SR-BI activation, probably via carboxyl-ester lipase (CEL). CEL is expressed by hepatocytes and co-localizes with SR-BI at the cell surface. It cooperates with SR-BI to hydrolyse HDL derived CE [30]. In addition, its activation by taurocholate stimulates selective CE uptake. This stimulation is independent of its hydrolysis activity as the uptake of hydrolysable cholesteryl-esters and non-hydrolysable cholesteryl-ethers is equally affected [31]. Therefore, bile acids seem to induce selective lipid uptake by CEL activation, although HDL endocytosis is decreased. In SR-BI deficient cells, these effects were abolished (Fig. 4), suggesting that SR-BI activation is necessary to increase selective CE uptake and in turn down-regulates HDL endocytosis upon bile-acid treatment.

Besides their extracellular effects on HDL endocytosis, we found that bile acids reduce HDL endocytosis also by transcriptional effects (Fig. 5). Comparable effects were found with CDCA as well as the non-steroidal FXR agonist GW4064, which suggests that these effects are FXR mediated. The concentrations of CDCA used here were 50 and 100 µM, which is in the range of physiologic conditions.

Reduced HDL endocytosis after FXR activation was still apparent in SR-BI deficient cells (Fig. 6) and was presumably mediated by impaired CD36 expression and function after bile acid treatment (Fig. 7). Like SR-BI, CD36 is a scavenger receptor with a broad spectrum of ligands including oxidized and native lipoproteins. CD36 was identified as a receptor mediating HDL endocytosis *in-vivo* and *in-vitro* [27]. The mechanism, how FXR activation represses CD36 expression, remains to be investigated. Recent reports suggest that FXR activation reduces CD36 expression in the murine liver and in macrophages [32], [33]. Besides activating gene expression, FXR can also directly act as a transcriptional repressor. For instance, hepatic lipase and apoA-I, which are both relevant to HDL metabolism, are repressed by FXR [34], [35].

When SR-BI levels were strongly reduced in HepG2 cells, there was still considerable residual HDL cell association apparent (compare Figs. 4 and 6). Other receptors such as the low affinity binding site under the control of F_1_-ATPase/P_2_Y_13_ as well as CD36 might account for this residual activity. In line, SR-BI does not seem to be the major factor determining hepatic HDL endocytosis [6], [10]. In contrast, SR-BI is the main receptor mediating selective lipid uptake from HDL. Our results show that SR-BI expression is unaltered after FXR activation (Fig. 6). Recent studies report that FXR activates SR-BI expression [24], [25], [36]. However, it was also found that FXR activation represses SR-BI by a mechanism comparable to the repression for Cyp7a1 [26]. The reasons for these discrepancies remain unknown. Due to unaltered SR-BI expression after CDCA or GW4064 treatment in our experiments, cholesteryl-ester uptake from HDL was unchanged. This resulted in an increase of calculated selective uptake, because HDL particle association was reduced (Fig. 6).

Altogether, our data have implications for the connection between HDL endocytosis and selective uptake. When HDL uptake in HepG2 cells was reduced either by extracellular or transcriptional mechanisms, no concomitant reduction in cholesteryl-ester uptake was observed. In contrast, selective CE uptake seemed to be differentially regulated. HDL endocytic trafficking is accompanied by lipid exchange [5], [37]. In addition, pharmacological interference with HDL endocytosis resulted in induced flux of HDL cholesterol from the plasma to the liver and enhanced biliary cholesterol secretion [38]. However, HDL endocytosis is no prerequisite for selective lipid uptake: liposomes containing purified SR-BI take up CE efficiently [39]. Moreover, different experimental approaches to block HDL endocytosis do not affect selective uptake [40], [41]. Consistently, our data presented here suggest that HDL endocytosis and selective CE uptake are not necessarily linked with each other. Indeed, *in-vivo* studies suggest that bile acids increase selective lipid uptake, thereby enhancing the clearance of HDL cholesterol from the plasma. Bile acid feeding lowers HDL cholesterol in mice [42]. Consistently, GW4064 administration decreases HDL cholesterol in mice [36] and the synthetic FXR agonist PX 20606 decreased plasma HDL levels in cynomolgus monkeys [43]. In contrast, FXR knockout mice have increased HDL cholesterol levels [23].

Taken together, our results indicate that bile acids reduce HDL endocytosis by transcriptional and non-transcriptional mechanisms. However, reduced HDL endocytosis is not accompanied by reduced cholesteryl-ester transfer.
